# Interim and end-treatment ^18^F-Fluorocholine PET/CT and bone scan in prostate cancer patients treated with Radium 223 dichloride

**DOI:** 10.1038/s41598-021-86759-1

**Published:** 2021-04-01

**Authors:** Ana María García Vicente, Mariano Amo-Salas, Javier Cassinello Espinosa, Roberto Gómez Díaz, Ángel Soriano Castrejón

**Affiliations:** 1 Nuclear Medicine Department, Hospital General Universitario, C/Obispo Rafael Torija S/N, 13005 Ciudad Real, Spain; 2 Mathematics Department, Universidad de Castilla-La Mancha, Ciudad Real, Spain; 3 Oncology Department, Hospital Universitario de Guadalajara, Guadalajara, Spain; 4 Oncology Department, Hospital General Universitario, Ciudad Real, Spain

**Keywords:** Prostate cancer, Molecular medicine

## Abstract

To assess the predictive and prognostic aim of interim and end-treatment ^18^F-fluorocholine PET/CT (FCH-PET/CT) and ^99m^Tc-methilen diphosphonate bone scintigraphy (BS) in patients with castration-resistant prostate cancer and bone metastases (CRPC-BM) treated with Radium 223 dichloride (^223^Ra). Prospective and multicentre ChoPET-Rad study including 82 patients with CRPC-BM. Baseline, after 3 (interim) and 6 doses (end-treatment) BS and FCH PET/CT were performed in patients who meet the study criteria. Clinical variables, imaging and clinical progression were obtained and their association with progression free survival (PFS), and overall survival (OS) was studied. Agreement between BS and FCH PET/CT response was assessed using Kappa (K) analysis. Median of PFS and OS was 3 and 16 months, respectively. Agreement between interim BS and FCH PET/CT was weak (K: 0.28; p = 0.004). No agreement was observed between end-treatment diagnostic studies. Interim and end-treatment FCH PET/CT were related to PFS (p = 0.011 and p < 0.001, respectively). Therapeutic failure and interim BS and FCH PET/CT showed association with OS (p < 0.001, p = 0.037 and p = 0.008, respectively). Interim and end-treatment FCH PET/CT were good predictors of biochemical progression in patients treated with ^223^Ra. Therapeutic failure and progression in interim BS or FCH PET/CT were adverse factors for OS.

## Introduction

Radium 223 dichloride (^223^Ra) is a targeted alpha therapy introduced into clinical practice for the treatment of patients with castration resistant prostate cancer with bone metastases (CRPC-BM)^1–4^.

In patients with CRPC-BM the lack of suitable biologic and imaging markers, able to guide in patient selection and response assessment, is a potential and challenging drawback for ^223^Ra as well as for other therapies. In clinical practice, monitoring of treatment with ^223^Ra is based on clinical and biochemical markers, being the role of imaging not well documented, with few works regarding to bone scintigraphy (BS) and computed tomography (CT), and only case reports for positron emission tomography/computed tomography using radiolabeled choline analogues (choline-PET/CT)^5–12^. This “evidence of absence” explains that most current guidelines [e.g. European Society of Medical Oncology, European Association of Urology and the American Urological Association] do not provide clear recommendations about diagnostic imaging assessment in clinical practice or only focused on clinical trials^3,4,13–15^.

Based on the higher sensitivity and specificity of choline-PET/CT, respect to standard BS, in detecting bone metastases and the potential for diagnosing extraosseous disease in the same exploration, some advantages respect to the BS in the response assessment should be expected^16–19^. However although the Prostate Cancer Radiographic Assessments for Detection of Advanced Recurrence II (RADAR II) group and the National Comprehensive Cancer Network (NCCN) guidelines recognize the use of choline-PET/CT in patients with biochemical recurrence, no recommendations are given regarding the response assessment^20–22^.

Based on: (1) the limited experience about the use of ^18^F-Fluorocholine (FCH) PET/CT, BS and CT in patients treated with ^223^Ra and (2) the reduced accuracy of BS and CT in the response assessment in patients with CRPC-BM a prospective and multicentre study was designed to assess the value of FCH PET/CT and BS in the response and prognosis assessment of patients treated with ^223^Ra, aiming to validate and introduce FCH PET/CT into the diagnostic management of patients with ^223^Ra indication.

## Material and methods

The present study (FCH PET/CT in the assessment of ^223^Ra treatment response, ChoPET-Rad) was designed as a prospective, multicentre (six hospitals) and non-randomized.

Ethical Committee of the coordinator centre (University General Hospital, Ciudad Real, Spain) where all the FCH PET/CT were performed approved the study (code: C/52/2016). Informed consent was obtained from all patients.

### Patients

Patients with CRPC-BM who fulfill all the inclusion criteria and none of the exclusion criteria for ^223^Ra treatment were consecutively included between January 2015 and January 2020.

The inclusion criteria for initiating ^223^Ra treatment were: (a) patients with CRPC with symptomatic bone metastases and a negative or inconclusive CT for visceral metastatic disease performed in the previous 6 weeks to request ^223^Ra treatment; (b) patients with a good bone marrow reserve that fulfills the hematologic criteria necessary to administer ^223^Ra and (c) Eastern Cooperative Oncology Group (ECOG) performance status of 0–1 (life expectancy greater than 6 months).

The exclusion criteria were: (a) patients who denied participating in the study or (b) who not fulfill any of the inclusion criteria or (c) were diagnosed of visceral or diffuse bone marrow involvement on baseline FCH PET/CT and/or BS^23^.

Patients scheduled to be treated with of ^223^Ra (55 KBq/kg, intravenously) in a 4-week cycle, for six cycles and maintained on androgen deprivation therapy.

### Clinical assessment

Each patient was clinically, hematological and biochemically evaluated before each ^223^Ra administration and bimonthly or monthly, after the last ^223^Ra administration, depending on the patient clinical status and the subsequent therapeutic lines. The clinical and biochemical variables were: Gleason, prostate specific antigen (PSA), alkaline phosphatase (AP), lactate dehydrogenase (LDH), time of evolution of prostate cancer, time of evolution of bone metastases and therapeutic line that ^223^Ra represented.

LDH and AP were considered as pathological when the values were higher than 333 U/L and 147 U/L, respectively.

The percent of change in PSA of each month determination during ^223^Ra administration with respect to baseline value was calculated in order to obtain the progression free survival (PFS).

Clinical progression was assessed following the RADAR II group recommendation^22^ using at least two of the following indicators: (1) convincing and consistent rise in PSA (defined as three consecutive rises, resulting in two 50% increases over the basal PSA value), (2) evidence of radiographic progression, or (3) worsening of the status performance or clinical symptoms while the patient is on therapy.

Thus at least two of three criteria (PSA progression, radiographic progression and clinical deterioration) should be fulfilled to stop ^223^Ra treatment. Finally the decision to stop treatment was addressed in multidisciplinary evaluation (oncologist and nuclear medicine physician) mostly after the third or fourth ^223^Ra treatment, based on early progression. Last follow-up was performed in June 2020.

Clinical end-points were: (a) treatment failure, defined as an incomplete ^223^Ra administration (less than six cycles) because of clinical progression of the disease or hematological toxicity and/or clinical impairment, (b) PFS, attending to PSA evolution, defined as three consecutive rises in PSA, resulting in two ≥ 50% increases over the basal PSA value and (c) overall survival (OS), defined as the elapsed time between the date of the start of ^223^Ra and the date of either death or the last follow-up.

For PSA, response was considered if a decrease in absolute PSA value of ≥ 30% between baseline PSA and interim (1 month later the third dose) or end-treatment (1 month later the sixth dose) was observed, defined as early of delayed response, respectively. Stability was considered for the rest of biochemical changes.

For AP and LDH, progression was defined as an increase of ≥ 25% from last available determination during treatment respect to baseline observed. Response was defined as a reduction of ≥ 30% from the baseline value. The rest of conditions were considered as stability.

### Imaging acquisition

FCH PET/CT and BS were performed, within a time interval of 4 weeks, before the first administration (baseline), before the fourth (interim) and after the sixth (end-treatment) ^223^Ra doses.

Bone scan was acquired 3 h after injection of 740 MBq of ^99m^Tc-methilen diphosphonate (MDP) in three reference centres. FCH PET/CT was performed in a unique reference hospital, 5–15 min after intravenous administration of 2–4 MBq/kg, in three-dimensional acquisition mode for 3 min per bed position, from skull to proximal legs. Low dose CT (120 kV, 80 mA) without contrast was performed for attenuation correction and as an anatomical map. The emission data was corrected for scatter, random coincidence events, and system dead time using the provided software^23^.

### Imaging evaluation

Two independent observers evaluated FCH PET/CT and BS. In case of discordances, a third observed reviewed the studies to reach a consensus.

Baseline FCH PET/CT and BS were visually evaluated establishing the BM extension following the criteria defined in a previous publication^23^.

Interim and end-treatment BS and FCH PET/CT were compared with respect to the previous one, assessing all the included anatomical areas (preferable axial and proximal third of extremities), in order to establish response (baseline and interim, respectively). The appearance of at least two new areas of increased uptake, osseous on BS and osseous and/or extraosseous on FCH PET/CT or a new lesion associated to an increase of the extent of BS or FCH uptake (at least 20% in the longest direction) in another one bone was considered as progression following the criteria for response formulated by the National Prostatic Cancer Treatment Group, formerly called the National Prostatic Cancer Project (NPCP)^24^.

In BS, complete response, lesion stability or partial response with a reduction by 50% in the number of increased areas of uptake were joined for statistical purposes and considered no progressive disease. In FCH PET/CT no progression was considered in cases of stability, partial response or complete response according to criteria of the European Organization for Research and Treatment of Cancer (EORTC)^25^.

### Statistical analysis

Statistical analysis was performed using SPSS software (v. 22). Quantitative variables were represented by mean and standard deviation and qualitative variables by frequency and percentage. Relation between qualitative variables was studied using Chi-squared Pearson test.

Cohen’s kappa coefficient was used to report the concordance regarding the response results between BS and FCH PET/CT, classifying the results as poor (< 0.20), weak (0.21–0.40), moderate (0.41–0.60), good (0.61–0.80) and very good (0.81–1.00).

Kaplan–Meier survival analysis and Cox regression were considered to study the prognostic factors of the overall survival (OS) and the progression free survival (PFS). The median follow-up was estimated using reverse Kaplan–Meier method.

The significance level was established at p < 0.05.

### Ethical approval

All procedures performed in studies involving human participants were in accordance with the ethical standards of the institutional and/or national research committee and with the 1964 Helsinki declaration and its later amendments or comparable ethical standards.

### Informed consent

Informed consent was obtained from all individual participants included in the study.

## Results

^223^Ra treatment was requested in 98 patients. Evaluation of the clinical and hematological conditions dismissed 16 cases: four for hematological constraints, five attending to FCH PET/CT results consisted on metastatic disease in three patients (lung, liver and brain involvement, respectively) and two with locorregional invasiveness (seminal vesicle invasion and bladder involvement, respectively), three for diffuse bone marrow involvement defined by BS and/or FCH PET/CT, one for a probably second primary tumor (hypernephroma) without a definite diagnosis, two for clinical deterioration with ECOG > 2 and one for medullar compression days previous to ^223^Ra initiation. Thus 82 patients were finally enrolled in the present study.

The median follow-up was 39 months with a median PFS and OS of 3 and 16 months, respectively.

Sixty-one out of 82 (74.4%) patients had received docetaxel treatment before study enrollment. ^223^Ra was considered as the first or second therapeutic line in only 23 patients (28%). Tables 1 and 2 show the patient and disease characteristics.

**Table 1:** Patient and tumor characteristics.

Baseline quantitative variables	Mean ± SD
Age (years)	72.41 ± 8.55
Baseline PSA (ng/ml)	213.00 ± 1006.55
Baseline AP (U/l)	188.96 ± 199.66
Baseline LDH (U/l)	434.02 ± 193.32

Time variables	Median (IQR)
Time of evolution of prostate cancer (months)	60 (32.75–108.00)
Time of evolution of metastases (months)	29 (20.00–49.50)
Time of castration resistance (months)	21 (12.00–40.50)

*SD* standard deviation, *AP* alkaline phosphatase, *LDH* lactate dehydrogenase, *PSA* prostate specific antigen, *IQR* interquartile range.

**Table 2:** Patients and tumour characteristics (qualitative and categorical variables).

Clinical characteristics	n	%
**Gleason**		
≤ 7	36	43.9
≥ 8	37	45.1
n.a.	9	11.0
**Biochemical characteristics**		
Baseline AP		
Pathological	30	36.6
Normal	40	48.8
n.a.	12	14.6
Baseline LDH		
Pathological	16	19.5
Normal	54	65.9
n.a.	12	85.4
**Extent of BM on baseline BS**		
1–5 lesions	19	23.2
6–20 lesions	28	34.1
> 20 lesions	28	34.1
Superscan	7	8.5
**Extent of BM on baseline FCH PET/CT**	69	
1–5 lesions	21	30.4
6–20 lesions	24	34.8
> 20 lesions	18	26.1
Superscan	6	8.7
**Extraosseous involvement on baseline FCH PET/CT**	69	
Yes	16	23.2
No	53	76.8
^**223**^**Ra line**		
First three lines	65	79.3
Following	17	20.7
**Docetaxel previous to **^**223**^**Ra**		
Yes	61	74.4
No	21	25.6
**Death during follow-up**		
Yes	63	76.8
No	19	23.2
**Therapeutic failure**		
Yes	48	58.5
No	34	41.5
**PSA progression during **^**223**^**Ra treatment**		
Yes	54	65.9
No	21	25.6
n.a.	7	8.5
Interim BS response	52	
Progression	21	40.4
No progression	31	59.6
**End-treatment BS response**	39	
Progression	11	28.2
No progression	28	71.8
**Interim FCH-PET/CT response**	50	
Progression	31	62.0
No progression	19	38.0
**End-treatment FCH-PET/CT response**	29	
Progression	17	58.6
No progression	12	41.4

*AP* alkaline phosphatase, *LDH* lactate dehydrogenase, *n.a*. not available, *n* number of patients, *BM* bone metastases, *BS* bone scan.

Two thirds of patients biochemically progress during ^223^Ra treatment (54, 65.9%). 40/54 (74.1%) of these progressions were during the first 3 months after ^223^Ra initiation (early progression). 48 out of 82 (58.5%) patients did not complete the six administrations of ^223^Ra (therapeutic failure), 43 for clinical progression (RADAR criteria) and five cases for other clinical reasons (one pulmonary thromboembolism, two hematological constraints and two clinical deterioration). On the other hand, three patients with clinical progression (RADAR criteria) completed the six doses of ^223^Ra treatment based on their limited therapeutic options and/or “low burden” progression on imaging, less than five small new lesions in non-visceral locations.

Only 13 patients experienced PSA response during ^223^Ra treatment, nine of them early responses that kept during treatment and the rest delayed ones.

Regarding the binary response (progression vs no progression) on imaging, progression was detected more frequently on interim and end-treatment FCH PET/CT, compared to BS (Tables 2 and 3). Regarding the pattern of progression on FCH PET/CT, all the cases were bone progression except in three cases with exclusively lymph node progression in interim and end-treatment evaluations. Visceral involvement (liver metastases on CT portion of FCH PET/CT with absent or minimal FCH uptake) was observed in six patients, five on interim evaluation and one on end-treatment FCH PET/CT. All the cases associated bone progression.

**Table 3:** Response results of interim and end-treatment bone scan and FCH PET/CT.

	Interim FCH PET/CT				
		PR	E	P	
Interim BS	PR	1	0	1	
	E	6	9	12	
	P	1	2	17	

	End-treatment FCH PET/CT				
		CR	PR	E	P
End-treatment BS	PR	1	0	1	1
	E	1	1	7	10
	P	0	0	1	5

*PR* partial response, *CR* complete response, *E* stable, *P* progression, *BS* bone scan, *FCH PET/CT* 18F-fluorocholine positron emission tomography/computed tomography.

Agreement between interim BS and FCH PET/CT was weak (K: 0.28; p = 0.004). No agreement was observed between end-treatment diagnostic studies (K: 0.13; p = 0.247). The distribution of the results is showed in Table 3.

Progression of AP and LDH was observed in 14/64 and 19/59 cases, respectively. Response of AP and LDH was detected in 18/64 and 9/59 cases, respectively. Stability was observed in the rest of cases, especially for LDH.

Baseline PSA and AP showed significant association with OS. For every 100 units or 10 units that PSA or AP increases, the risk of death raises by 2% (HR = 1.024; p = 0.025 and HR = 1.026; p < 0.001, respectively).

LDH progression showed significant association with PFS in Cox-analysis (HR: 2.126; p = 0.020).

**Figure 1:**
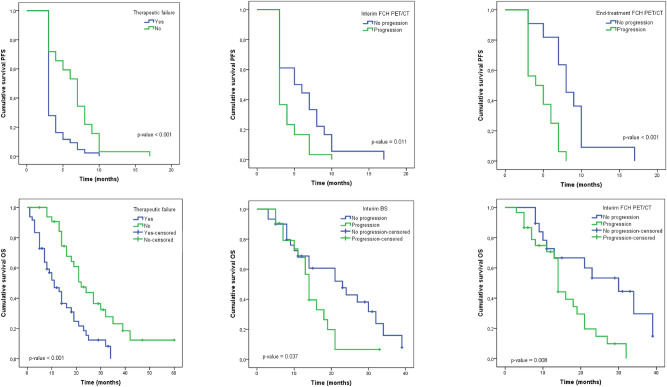
Upper row: Log Rank results of therapeutic failure (**A**), interim FCH PET/CT (**B**) and end-treatment FCH PET/CT (**C**) in their associations with PFS. Lower row: Log Rank results of therapeutic failure (**D**), interim BS (**E**) and interim FCH PET/CT (**F**) in their associations with OS.

Significant association of therapeutic failure with PFS and OS were found using Cox regression and Kaplan–Meier analysis. Interim and end-treatment FCH PET/CT results showed significant association with PFS whereas interim BS and FCH PET/CT results showed significant association with OS. The results are detailed on Tables 4 and 5. Figure 1 shows graphically the most relevant results.

**Table 4:** Long rank tests and Cox regression results of association of clinical and imaging response variables with PFS.

Variables	Median	Mean ± SD	χ^2^	P valor	HR (95% CI)	P valor
**Therapeutic failure**						
Yes	3	3.74 ± 0.24	18.64	< 0.001	2.26 (1.37–3.74)	0.001
No	7	6.44 ± 0.56				
**Gleason**						
≥ 8	3	4.63 ± 0.53	0.05	0.829	1.04 (0.64–1.69)	0.872
≤ 7	4	4.91 ± 0.39				
**Docetaxel previous to **^**223**^**Ra**						
Yes	3	4.98 ± 0.39	0.23	0.629	0.91 (0.55–1.51)	0.714
No	3	4.67 ± 0.48				
^**223**^**Ra first three lines**						
Yes	3	4.77 ± 0.36	0.34	0.562	1.14 (0.64–2.01)	0.656
No	5	5.40 ± 0.64				
**Interim BS**						
No P	4	5.33 ± 0.58	2.24	0.134	0.72 (0.40–1.28)	0.265
P	3	4.05 ± 0.45				
**Interim FCH PET/CT**						
No P	5	6.28 ± 0.45	6.49	0.011	0.54 (0.29–1.02)	0.057
P	3	4.03 ± 0.33				
**End-treatment BS**						
No P	6	6.65 ± 0.67	2.27	0.132	0.60 (0.28–1.29)	0.193
P	7	5.54 ± 0.62				
**End-treatment FCH PET/CT**						
No P	8	8.54 ± 1.07	12.56	< 0.001	0.23 (0.08–0.62)	0.004
P	4	4.75 ± 0.46				

*HR* hazard ratio, *PFS* progression free survival, *χ*^*2*^ Chi-squared, *P* progression, *no P* no progression, *SD* standarddeviation, *CI* confidence interval, *BS* bone scan, *FCH PET/CT* 18F-fluorocholine positron emission tomography/computed tomography.

**Table 5:** Long rank tests and Cox regression results of association of clinical and imaging response variables with OS.

Variables	Median	Mean ± SD	χ^2^	P valor	HR (95% CI)	P valor
**Therapeutic failure**						
Yes	11	13.69 ± 1.55	13.92	< 0.001	2.65 (1.54–4.59)	< 0.001
No	22	27.18 ± 2.46				
**Gleason**						
≥ 8	19	21.55 ± 2.71	0.30	0.586	0.86 (0.50–1.49)	0.597
≤ 7	18	19.83 ± 2.46				
**Docetaxel previous to **^**223**^**Ra**						
Yes	16	20.20 ± 2.30	0.11	0.737	0.91 (0.51–1.62)	0.745
No	14	17.83 ± 1.98				
^**223**^**Ra first two lines**						
Yes	19	22.69 ± 2.36	1.70	0.193	1.14 (0.64–2.01)	0.656
No	14	18.70 ± 2.38				
**Interim BS**						
No P	23	22.01 ± 2.42	4.37	0.037	0.49 (0.24–0.99)	0.047
P	14	4.741 ± 1.65				
**Interim FCH PET/CT**						
No P	30	25.49 ± 3.14	7.03	0.008	0.36 (0.16–0.81)	0.013
P	14	15.90 ± 1.68				
**End-treatment BS**						
No P	24	28.26 ± 3.43	3.32	0.070	0.48 (0.21–1.09)	0.080
P	16	18.45 ± 2.24				
**End-treatment FCH PET/CT**						
No P	24	23.52 ± 3.89	0.06	0.811	1.11 (0.45–2.73)	0.816
P	21	24.98 ± 3.24				

*HR* hazard ratio, *PFS* progression free survival, *χ*^*2*^ Chi-squared, *P* progression, *no P* no progression, *SD* standard deviation, *CI* confidence interval, *BS* bone scan, *FCH PET/CT* 18F-fluorocholine positron emission tomography/computed tomography.

In multivariate analysis, only therapeutic failure and interim FCH PET/CT showed association with PFS (p = 0.047 and p = 0.078, respectively). Thus, a patient with therapeutic failure or progression in interim FCH PET/CT had 1.938 (1.008, 3.727) or 1.789 (0.938, 3.413) times more risk of PSA progression than patients without the cited conditions. On the other hand, the independent and significant variables associated with OS were therapeutic failure (p = 0.034) and baseline AP (p = 0.001).

**Figure 2:**
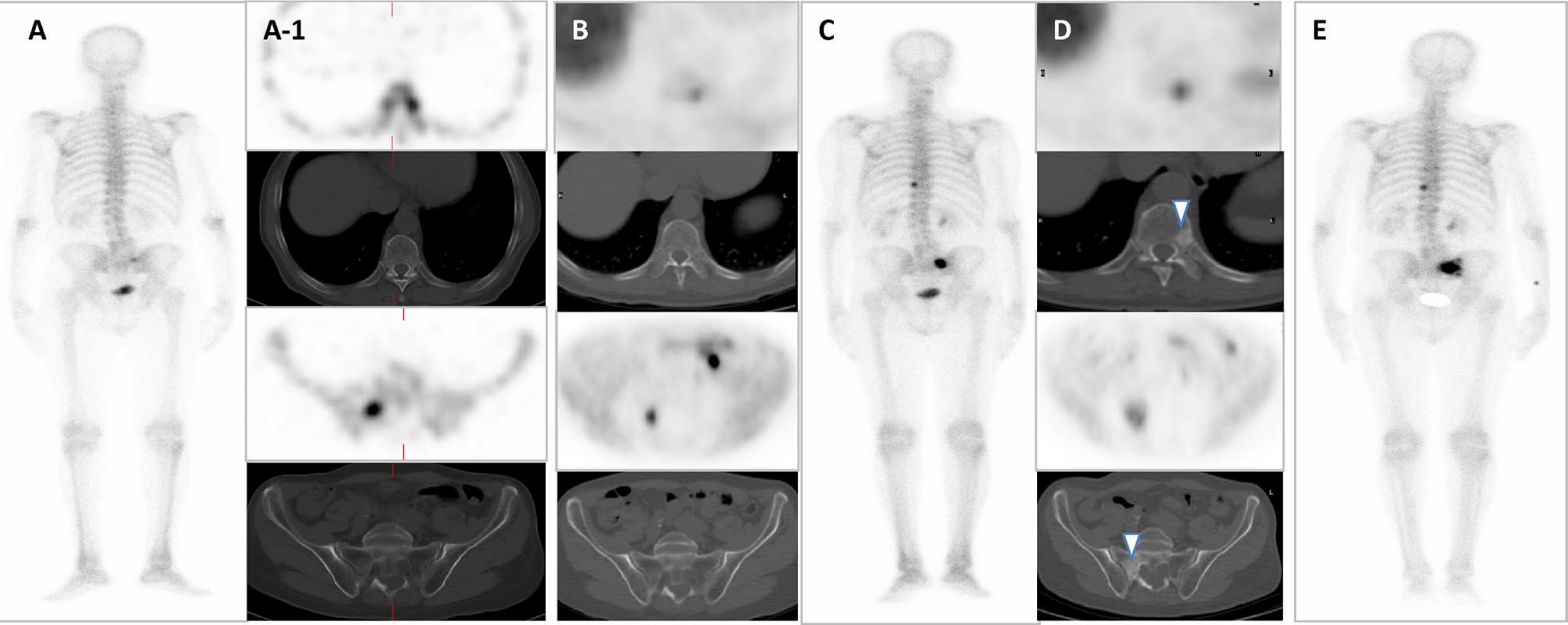
Patient ≠ 1. Baseline total body BS (**A**) and SPECT/CT (**A-1**) show oligometastatic disease in dorsal spine and right sacrum. Baseline FCH PET/CT (**B**) shows the same pathological locations, without alterations in bone density on CT. Interim total body BS (**C**) showing stability in number of lesions with an increase in osteoblastic activity. Stability regarding the number of lesions on interim FCH PET/CT (**D**) with an increase of density in CT portion (arrowheads). Clinical progression was diagnosed with clinical impairment plus PSA progression, with an increase in size of known lesions on end-treatment BS (**E**). 74 years old man, Gleason 9, ^223^Ra in third line, baseline PSA: 32 ng/ml. PFS and OS of 3 and 23 months, respectively.

**Figure 3:**
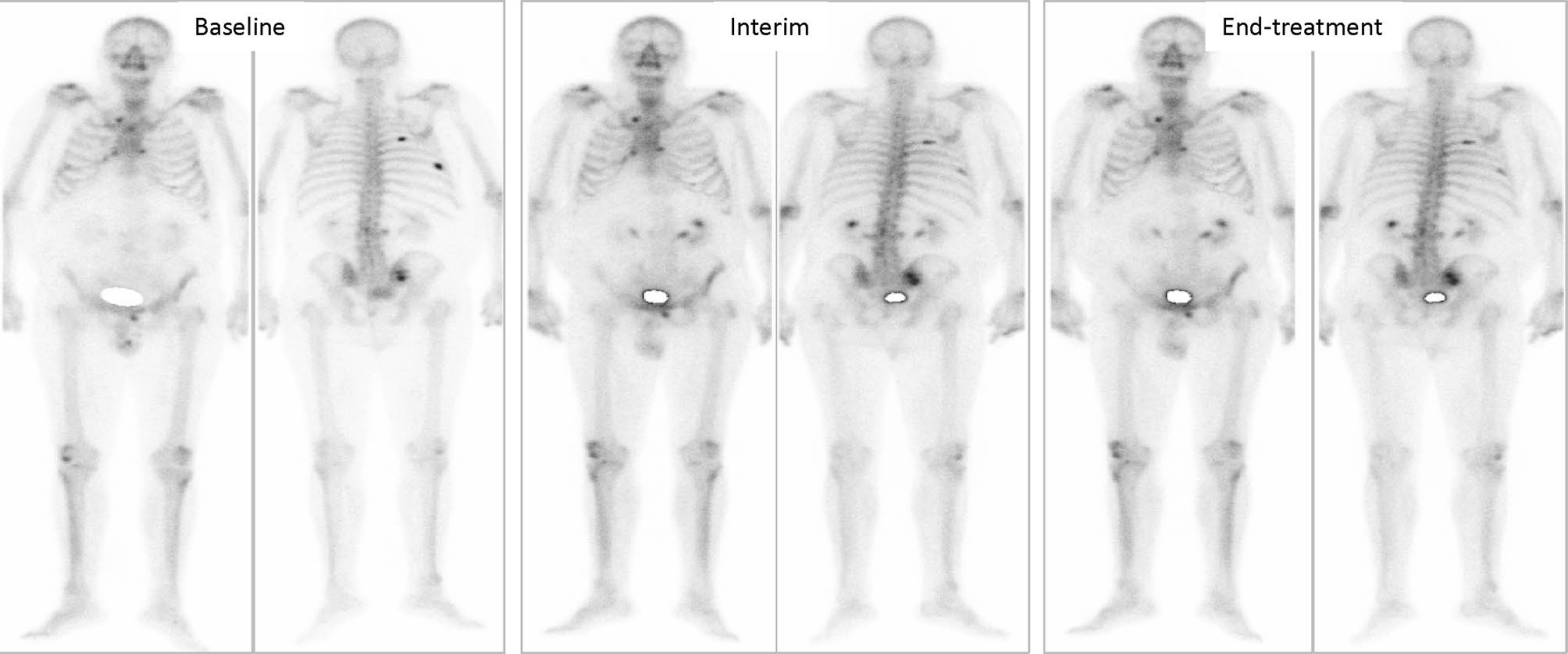
Patient ≠ 2. Baseline BS showing stability on interim and end-treatment BS in a oligometastasic disease 81 years old man, Gleason 6, ^223^Ra in first line, baseline PSA: 15 ng/ml. PFS and OS of 10 and 34 months, respectively.

**Figure 4:**
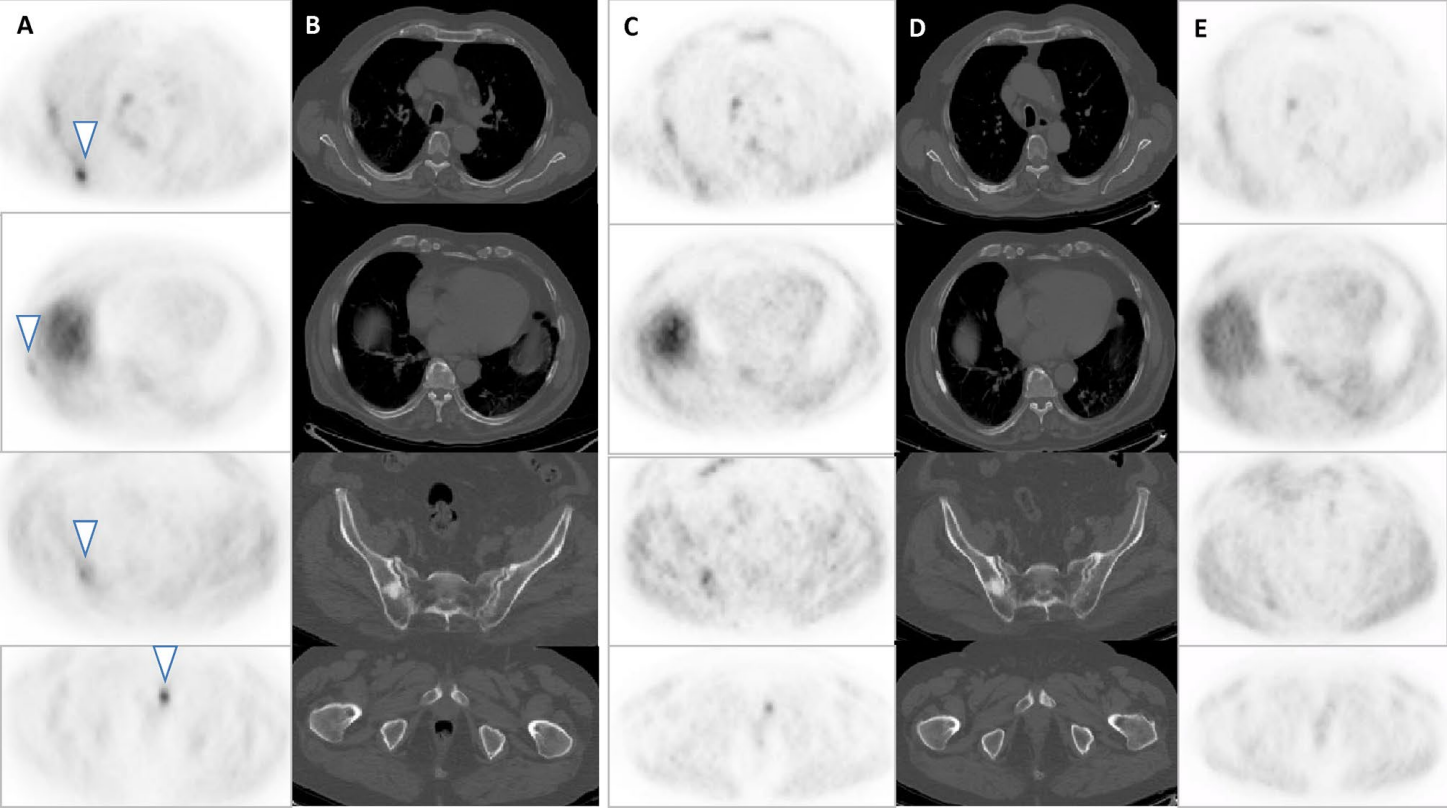
Patient ≠ 2. Axial slices of baseline FCH PET/CT (**A**, **B**) showing four lesions with uptake and blastic reaction (arrow-heads). Axial slices of interim FCH PET/CT (**C**, **D**) show stability in lesion number, with reduction of metabolism. Axial slices of end-treatment FCH PET portion (**E**) show a reduction of uptake with minimal visual detection of lesions.

**Figure 5:**
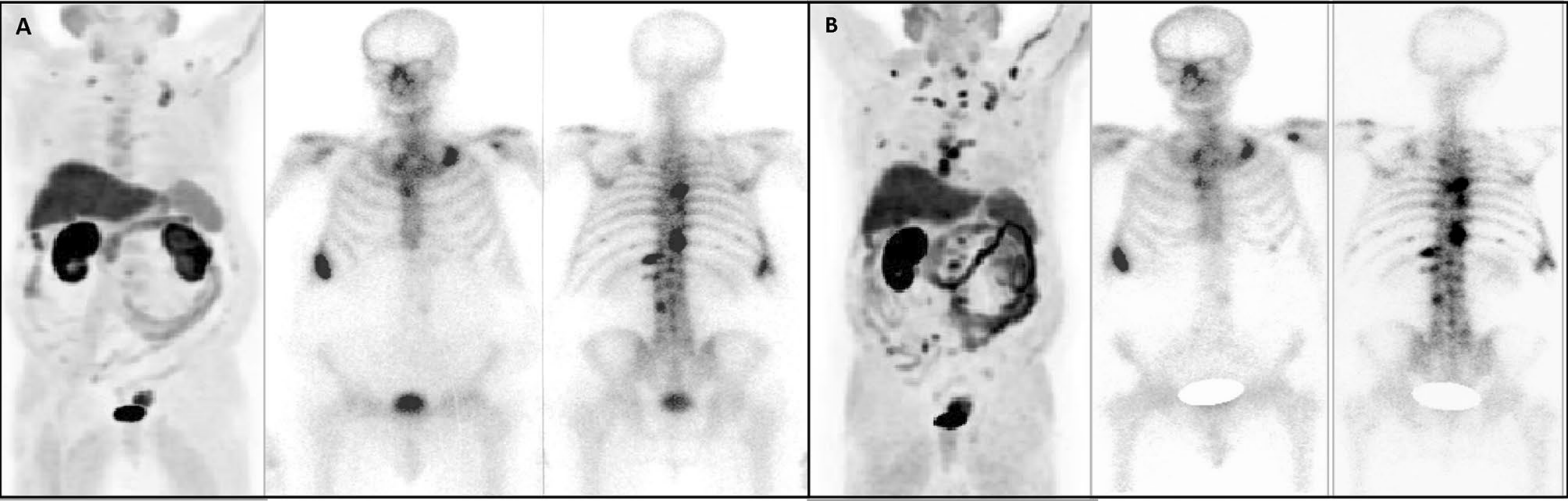
Patient ≠ 3. Baseline (**A**) and interim (**B**) series of FCH PET/CT (left images) and BS (central and right images) show polimetastatic osteoblastic disease. Interim BS was stable with respect to baseline study, whereas progression was detected on interim FCH PET/CT. 82 years old man, Gleason 9, ^223^Ra in third line, baseline PSA: 59 ng/ml. PFS of 3 months, under follow-up for OS.

**Figure 6:**
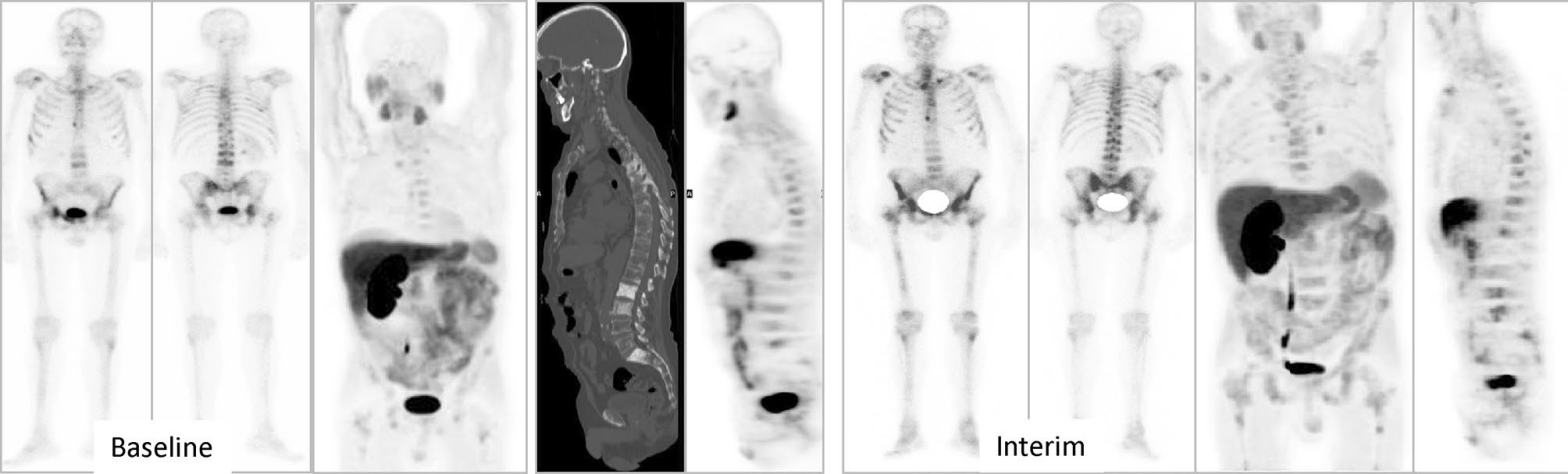
Patient ≠ 4. Baseline BS and FCH PET/CT shows polimetastatic disease with progression on interim images. 66 years old man, Gleason 9, ^223^Ra in second line, baseline PSA: 57 ng/ml. PFS and OS of 3 and 14 months, respectively.

Some representative cases are showed on Figs. 2, 3, 4, 5 and 6.

## Discussion

Regular monitoring of bone metastases for men with CRPC, using conventional imaging (CT and/or BS), although recommended, is notoriously difficult, that explains the use of “clinical benefit”, in common daily practice, as the best evidence of response. However the combined use of clinical, biochemical and imaging assessment is of paramount importance to a correct monitoring for treatments with survival impact as ^223^Ra, as we considered in the present work.

Prostatic specific antigen behavior during ^223^Ra treatment is not well known based on the limited experience. Reductions of PSA levels up to 18% have been associated to the cytotoxic effect of ^223^Ra inhibiting tumor-induced osteoblastic bone growth and suppressing bone metabolic activity^7,26–29^. On the other hand, PSA rise during treatment with ^223^Ra has been reported and attributed to various reasons: (a) progression of metastatic soft tissue injuries no involved in the treatment, (b) bone tumor lysis, (c) bone progression, (d) non androgen dependent mechanism of action of ^223^Ra or (e) flare^21,22,30–36^.

Based on the previously referred limitations we decided to use a very “restrictive” criterion for PSA progression, just only to use it as reference to compare with our imaging results in response assessment (BS and FCH PET/CT). This criterion practically rules out the misinterpretation of progressive disease as “flare” based on a steadily rising of PSA during ^223^Ra treatment less likely represented a flare phenomenon^37^.

On the other hand, AP and LDH can be useful in cases of discordant results between PSA values and clinical evaluation during ^223^Ra treatment^38^. A stability of AP and LDH was found in 32/64 and 31/59 cases, respectively. However LDH, contrary to AP, showed association with PFS. Thus, patients with LDH progression had a double risk to progress compared to the rest of patients (p = 0.020).

Previous works and current clinical guidelines are pragmatic about needing to assess response using diagnostic imaging, explaining the poorly defined use and interpretation criteria^5,6,21,39^. However guidelines, focused on imaging, state that imaging should be used when therapeutic selection could be affected by results [i.e., (1) when considering starting therapy, (2) before changing therapy to establish a new baseline, and (3) when significant deterioration of the general condition of the patient or consistent and convincing biochemical progression is identified^22,40,41^.

Regarding to the election of imaging technique, radiological evaluation using CT has recently been suggested by the European Expert Consensus Panel as the ideal method for predicting response to treatment in patients with CRPC-BM, even with a proven poor sensitivity and specificity^42–44^.

In the ^223^Ra setting, interim evaluation is recommended in patients with aggressive disease (high Gleason score at diagnosis or PSA doubling time < 6 months before starting ^223^Ra) in order to rule out visceral metastases, using the same technique as baseline assessment. In addition, in patients presenting worsening pain after the first three cycles, increase in AP level or low PSA doubling time revealing a suspicion of progression of disease, imaging evaluation should be repeated^45^. However, with BS, there are not specific criteria for the positive identification of benefit or response apart from its inherent limitations on the response evaluation of diffuse metastatic bone disease or malignant “superscan” and flare phenomenon^11,44,46–48^.

The scarcely literature has reported variable imaging features after ^223^Ra treatment. In relation to BS, paradoxical response has been documented with a decrease in osteoblastic activity in existing lesions joined to the appearance of new metastatic lesions^30,46,49^. On the other hand, Keizman et al.^11^, reported stable disease using BS at 3 and 6 months in 74% and 94%, respectively.

Despite the most recent evidence that support the choice of choline analogues PET/CT as the preferred Nuclear Medicine imaging modality for treatment monitoring of metastatic CRPC, rather than BS, ^18^NaF or ^18^F-fluorodeoxiglucose-(^18^F-FDG) PET/CT, the proper use of choline PET/CT is not yet established by guidelines^4,50,51^.

In the present study, imaging data from BS and FCH PET/CT before, during and after therapy were used to monitor response in patients treated with ^223^Ra. For the establishment of metabolic progressive disease using FCH PET/CT, in order to exclude the possibility of flare response, we used the same criteria that BS instead of semiquantitative EORTC criteria. We observed a weak and null agreement between interim and end-treatment BS and FCH PET/CT, respectively. Thus interim and end-treatment FCH PET/CT was superior to BS in the PFS definition, and thus more valid to monitor ^223^Ra treatment response.

Other authors described, using FCH, ^18^NaF or ^68^ Ga-prostate specific membrane antigen (^68^ Ga-PSMA) PET/CT, acute metabolic changes in large metastatic deposits after ^223^Ra treatment, with a dramatic drop of uptake in responders accompanied by a reduction of PSA and AP^8,9,46,52,53^. These results can be explained based on FCH and ^68^Ga-PSMA represents the tumoricidal effect of ^223^Ra.

Recognizing the progression is crucial to move to other more effective therapies and to avoid toxicities. Thus, the only possible purpose of performing an imaging technique is to monitor progression. In this setting, choline-PET/CT can evaluate disease progression when discordances between biochemical, clinical and imaging response, using more recognized techniques as BS and CT, exist^9,15^.

In patients treated with ^223^Ra, progression has been reported using BS in up to 28%, and soft-tissue progression on CT, including visceral disease, in up to 50% of cases^5,10–12^. Our median PFS (3 months) was lower than the previously described using other therapies in earlier lines^54–58^. This fact might be explained, first by the cross-resistance phenomenon (more of 2/3 of our patients underwent previous docetaxel and unless other line previous to ^223^Ra), and second, by our close and exhaustive follow-up performed using the triple assessment (clinical, BS plus FCH PET/CT and PSA)^59^. Keizman et al.^11^ found similar results, with a median radiological PFS of 4.8 months, documenting extraskeletal radiological progression on CT scans in 46% with an uncommon bone progression (6%).

In our opinion, a significant deterioration of the general condition of the patient, as main criteria to perform imaging and check biomarkers as current guidelines recommend^32,39,40^, is a restrictive criteria that limits the early detection of progression.

Guidelines and consensus state that treatments with a proven survival benefit should not be stopped for PSA progression alone (in the absence of radiographic or clinical progression) and although symptomatic or radiographic progression is a more reliable trigger for either therapeutic layering or change, the use of at least two of three criteria (PSA progression, radiographic progression and clinical deterioration) should be fulfilled to stop treatment^15,21,22^. Based on that, in the present work, clinical and/or radiological progression was required, in association with PSA progression, to stop ^223^Ra treatment, although all the cases were discussed on multidisciplinary team. In fact, the strong association of therapeutic failure with PFS was due to the direct dependence between these two variables, explained by progression determined stopping ^223^Ra treatment.

The present study has some drawbacks. The relatively small population in end-treatment evaluation by imaging, based on the high rate of progressive disease that promoted stopping ^223^Ra treatment, probably limited the statistical capability of our results. In addition the multicentre nature limited obtaining some biochemical parameters.

Regarding the strengths, this is the first documented evidence of ^223^Ra response and prognostic assessment using FCH PET/CT and BS in a prospective and parallel evaluation, showing the usefulness of interim FCH PET/CT and baseline AP, as independent predictors of PFS and OS in multivariate analysis, apart from therapeutic failure.

## Conclusion

In patients treated with ^223^Ra, interim and end-treatment FCH PET/CT were good predictors of biochemical and clinical progression. Moreover interim FCH PET/CT and BS, joined to therapeutic failure, offered prognostic information.

The obtained results define the potential value of FCH PET/CT, with respect to BS, to monitor ^223^Ra response and guide treatment discontinuation in a safe and effective way.
